# Real-World Evidence of Tirzepatide in Indian Adults With Type 2 Diabetes: Significant Early Glycemic and Cardiometabolic Benefits

**DOI:** 10.7759/cureus.102866

**Published:** 2026-02-02

**Authors:** Kumar Prafull Chandra, Mukulesh Gupta, Rajiv Awasthi, Arunkumar R Pande, Nitin Gupta, Dinesh Kumar, Vivek Agarwal, Tanusree Gupta, Shally Awasthi, Harshita Lachhwani

**Affiliations:** 1 General Medicine and Diabetes Care, Chandra Diabetes Clinic, Lucknow, IND; 2 Diabetes and Endocrinology, Udyan Health Care, Lucknow, IND; 3 General Medicine, Prarthana Clinic and Diabetes Care Centre, Lucknow, IND; 4 Endocrinology, Lucknow Diabetes Endocrine and Thyroid Clinic, Lucknow, IND; 5 Endocrinology, Lucknow Hormone Center, Lucknow, IND; 6 General Medicine, Harsha Clinic, Lucknow, IND; 7 Endocrinology, RR Diabetes and Heart Care Centre, Lucknow, IND; 8 Obstetrics and Gynaecology, Udyan Health Care, Lucknow, IND; 9 Pediatric, King George's Medical College, Lucknow, IND

**Keywords:** cardiometabolic risk, glp1ra and gip, glycated hemoglobin, incretin, india, real world evidence, tirzepatide, type 2 diabetes, weight loss

## Abstract

Background: Tirzepatide, a novel dual GIP/GLP-1 receptor agonist, has demonstrated superior glycemic efficacy in randomized clinical trials. However, real-world data in Indian populations remain limited.

Objective: The primary objective of this multicenter retrospective study was to evaluate the change in glycated hemoglobin (HbA1c) at 12 weeks (three months) following initiation of tirzepatide in Indian adults with type 2 diabetes mellitus treated in routine clinical practice. Secondary objectives included assessment of changes in body weight, blood pressure, lipid parameters, hepatic fibrosis index (FIB-4), and evaluation of tolerability and treatment persistence during follow-up.

Methods: This retrospective multicenter study included 71 adults with type 2 diabetes who completed three months of tirzepatide therapy across seven centers in Lucknow, Uttar Pradesh. The primary outcome was the change in HbA1c. Secondary outcomes included body weight, blood pressure, lipid parameters, liver enzymes, FIB-4 index, and evaluation of tolerability and treatment persistence.

Results: Mean HbA1c decreased significantly from 8.7 ± 1.4% to 6.9 ± 1.0% (mean change −1.8 ± 1.2%, p<0.001), with 59.2% achieving HbA1c <7% at three months. Mean body weight declined by 4.7 ± 3.2 kg (5.7%, p<0.001). Significant improvements were observed in systolic blood pressure (−5.6 mmHg), total cholesterol (−16.6 mg/dL), LDL-cholesterol (−12.2 mg/dL), HDL-cholesterol (+2.6 mg/dL), triglycerides (−46.4 mg/dL), and liver enzymes (all p<0.001). FIB-4 index decreased from 1.24 ± 0.68 to 1.08 ± 0.54 (p=0.004). Gastrointestinal adverse events occurred in 59.2% of patients, predominantly mild to moderate. The treatment discontinuation rate was 22.2%, mainly due to gastrointestinal intolerance (36%) and financial constraints (32%).

Conclusion: In routine clinical practice, tirzepatide was associated with substantial glycemic improvement and meaningful weight loss at 12 weeks, along with favorable changes in cardiometabolic risk factors (blood pressure, lipids, hepatic indices). While the safety profile was consistent with global clinical trial data, real-world treatment persistence was significantly influenced by tolerability and affordability, highlighting important context-specific barriers in the Indian healthcare setting. Cost remains a key barrier to long-term use.

## Introduction

Type 2 diabetes mellitus (T2DM) represents a major global public health challenge, characterized by progressive insulin resistance, impaired pancreatic beta-cell function, and chronic hyperglycemia leading to significant morbidity and mortality [1]. India bears a disproportionate burden of this epidemic, with an estimated 101 million adults currently living with diabetes, making it the second-largest diabetes population globally [2]. The prevalence is projected to increase substantially over the coming decades, driven by rapid urbanization, dietary transitions, sedentary lifestyles, and genetic predisposition unique to South Asian populations [3].

Effective management of T2DM requires not only achieving optimal glycemic control but also addressing associated metabolic complications including obesity, dyslipidemia, hypertension, and cardiovascular risk factors [4]. Traditional therapeutic approaches have focused primarily on glucose lowering through various mechanisms, yet the multifaceted nature of the disease demands more comprehensive interventions that can simultaneously target multiple pathophysiological abnormalities [5].

The therapeutic landscape for T2DM has evolved considerably over the past two decades, with the emergence of incretin-based therapies representing a paradigm shift in diabetes management. Glucagon-like peptide-1 receptor agonists (GLP-1 RAs) have demonstrated remarkable efficacy in improving glycemic control, promoting weight loss, and reducing cardiovascular events [6,7]. However, glucose-dependent insulinotropic polypeptide (GIP), another incretin hormone, was historically considered to have limited therapeutic potential in T2DM due to the observation that its insulinotropic effects appeared to be diminished in individuals with diabetes [8].

Recent advances in our understanding of incretin physiology have challenged this assumption, revealing that the co-activation of both GIP and GLP-1 receptors may produce synergistic metabolic benefits. This scientific insight led to the development of tirzepatide, a novel once-weekly injectable dual glucose-dependent insulinotropic polypeptide (GIP) and glucagon-like peptide-1 (GLP-1) receptor agonist. Tirzepatide appears to reduce chronic low-grade inflammation and markers of oxidative stress through a combination of direct receptor signaling in metabolic tissues (GLP-1R and GIPR), favorable adipose-immune remodeling, improved lipid handling and hepatic fat reduction, enhanced mitochondrial function, and indirect effects from weight loss and improved glycaemia. Beyond its dual glucose-dependent insulinotropic polypeptide (GIP) and GLP-1 receptor agonism, tirzepatide exerts additional metabolic effects that may contribute to its clinical efficacy. Experimental and clinical data suggest that tirzepatide reduces chronic low-grade inflammation through modulation of adipose tissue macrophage activity and inflammatory cytokine signaling. Furthermore, improvements in mitochondrial efficiency and reductions in oxidative stress have been observed, potentially contributing to enhanced insulin sensitivity and hepatic metabolic function [9]. Tirzepatide represents the first medication in its class to simultaneously target both incretin pathways, offering a unique mechanism of action that enhances glucose-dependent insulin secretion, suppresses inappropriate glucagon release, delays gastric emptying, and increases satiety, collectively contributing to superior glycemic control and substantial weight reduction [10]. 

The efficacy and safety of tirzepatide have been extensively evaluated in the landmark SURPASS clinical trial program, which demonstrated its superiority over both conventional GLP-1 receptor agonists and basal insulin in achieving glycemic targets and promoting weight loss [11-14]. In the SURPASS-2 trial, tirzepatide demonstrated superior reductions in HbA1c and body weight compared to semaglutide 1 mg, achieving mean HbA1c reductions ranging from 2.01% to 2.46% across different doses (5 mg, 10 mg, and 15 mg) compared to 1.86% with semaglutide [13]. Weight reductions with tirzepatide ranged from 7.6 kg to 11.2 kg compared to 5.7 kg with semaglutide. These impressive results led to the approval of tirzepatide by the U.S. Food and Drug Administration (FDA) in May 2022 under the trade name Mounjaro for the treatment of adults with T2DM, and subsequently by regulatory authorities in multiple countries, including India [15].

Despite the robust evidence from randomized controlled trials conducted primarily in Western populations, there remains a paucity of real-world data on the effectiveness and safety of tirzepatide in the Indian population. South Asians, including Indians, exhibit distinct metabolic characteristics compared to other ethnic groups, including greater insulin resistance at lower body mass indices, higher visceral adiposity, reduced beta-cell reserve, and an earlier age of diabetes onset [16,17]. These population-specific differences in disease phenotype, along with variations in dietary patterns, healthcare infrastructure, medication adherence, and socioeconomic factors, underscore the importance of evaluating novel therapeutic agents within the local context [18]. Dual GIP/GLP-1 receptor agonism may be particularly advantageous in South Asians, who exhibit a "thin-fat" phenotype characterized by excess visceral adiposity, insulin resistance, and reduced beta-cell reserve despite lower BMI [19]. 

Real-world evidence complements data from clinical trials by providing insights into medication performance in routine clinical practice, where patient populations are more heterogeneous, comorbidities are more prevalent, and treatment protocols may differ from controlled study settings [20]. Such evidence is particularly valuable for informing clinical decision-making, optimizing treatment strategies, and developing region-specific practice guidelines that reflect the realities of local healthcare delivery [21].

The present study was therefore undertaken to evaluate the clinical outcomes at 12 weeks (three months) associated with tirzepatide initiation in Indian adults with T2DM within a real-world, multicenter setting. Specifically, this retrospective observational study aims to assess changes in key metabolic parameters, including glycemic control (HbA1c, fasting plasma glucose, postprandial glucose), body weight and anthropometric measurements, lipid profile, liver function, blood pressure, and renal function following three months of tirzepatide therapy. Additionally, the study evaluates the tolerability and treatment persistence of tirzepatide in this population, documenting adverse events and treatment discontinuations. By providing region-specific data on the effectiveness and safety of this novel dual incretin agonist, this study seeks to contribute valuable insights that may inform evidence-based clinical practice and optimize diabetes care for the Indian population.

## Materials and methods

Study design and setting

This was a multicenter, retrospective observational study conducted across seven secondary-care diabetes clinics in an urban Indian setting. Data were extracted from electronic medical records of adults with type 2 diabetes initiated on tirzepatide as part of routine clinical care. The study was designed in accordance with the Strengthening the Reporting of Observational Studies in Epidemiology (STROBE) guidelines for observational research [22]. The research protocol (CDC/TZP-RWD/2025/001, Version 01, dated July 3, 2025) received approval from the Institutional Human Ethics Committee, Udyaan Health Care, Lucknow, Uttar Pradesh (Registration Number: ECR/1300/Inst/UP/2019) prior to data collection and analysis [23]. The study period started after receiving approval from the Ethics Committee in July 2025, with the collection of data from patients who had been started on injection tirzepatide until July 2025, through the completion of data collection (November 15, 2025).

The study was conducted across seven specialized diabetes care centers located within Lucknow city, Uttar Pradesh, North India: (1) Chandra Diabetes Clinic, Gomtinagar; (2) Prarthana Clinic & Diabetes Care Centre, New Hyderabad; (3) Udyaan Health Care, Eldeco; (4) Harsha Clinic & Diabetes Centre, Alambag; (5) Lucknow Endocrine Diabetes and Thyroid Clinic, Gomtinagar; (6) RR Diabetes and Heart Care Centre, Gomtinagar; and (7) Lucknow Hormone Centre, Vikasnagar. All participating centers are secondary care facilities specializing in diabetes management, equipped with appropriate diagnostic and therapeutic facilities, and staffed by experienced diabetologists. The study period extended from the date of first tirzepatide initiation (April 2025) through the completion of data collection (November 15, 2025).

Study population and participant selection

The study population comprised adult patients with type 2 diabetes mellitus who were initiated on tirzepatide therapy as part of routine clinical care at the participating centers. Patients were eligible for inclusion if they met the following criteria: (1) age ≥18 years; (2) confirmed diagnosis of type 2 diabetes mellitus according to American Diabetes Association criteria [1]; (3) Indian nationality; (4) initiation of tirzepatide therapy within six months prior to data collection; and (5) availability of complete baseline clinical and laboratory data along with at least one follow-up assessment at three months post-initiation. Patients were excluded if they had: (1) type 1 diabetes mellitus or other specific types of diabetes (secondary diabetes, gestational diabetes, maturity-onset diabetes of the young); (2) pregnancy or lactation at the time of tirzepatide initiation; (3) concurrent participation in any interventional clinical trial; (4) major endocrine disorders including Cushing's syndrome or acromegaly that could significantly affect metabolic parameters; or (5) incomplete or inadequately documented medical records precluding meaningful analysis.

All consecutive patients meeting the eligibility criteria during the study period were included to minimize selection bias. Given the retrospective observational nature of this study utilizing existing clinical data, the need for prospective informed consent was waived by the ethics committee. However, all patients had previously provided general consent for use of their de-identified clinical data for research purposes at the time of registration at the participating centers. The study involved no additional interventions beyond standard clinical care, and treatment decisions were made independently of the research protocol by the treating physicians based on clinical judgment and established guidelines. No financial remuneration or other incentives were provided for study participation.

Data collection and variable definitions

Data were extracted by trained data custodians using a standardized case report form. Double data entry and formal source-data verification were not performed, which may introduce information bias and is acknowledged as a study limitation. All patient-identifying information was removed during the data extraction process, and each participant was assigned a unique study identification number comprising a site code and sequential patient number to maintain confidentiality throughout the analysis phase. 

Baseline demographic and clinical variables collected included: age (years), sex (male/female), date of birth, duration of diabetes (years from initial diagnosis), height (centimeters), body weight (kilograms), body mass index (BMI, calculated as weight in kilograms divided by height in meters squared), and waist circumference (centimeters). Comorbidity data were recorded, including the presence and duration of hypertension, dyslipidemia, thyroid disorders, coronary artery disease (CAD), chronic kidney disease (CKD), and chronic liver disease/metabolic dysfunction-associated steatotic liver disease (CLD/MASLD). Information regarding prior and concurrent antidiabetic medications was systematically documented.

Tirzepatide initiation data included the date of first administration and the initial dose prescribed (2.5 mg, 5 mg, 7.5 mg, 10 mg, 12.5 mg, or 15 mg subcutaneously once weekly). Dose initiation and escalation were determined by treating physicians based on routine clinical judgment. In general, dose escalation from the starter dose to 5 mg was considered after four weeks in patients with suboptimal glycemic response, inadequate weight reduction, and acceptable gastrointestinal tolerability. Dose escalation was not protocol-mandated and reflects real-world prescribing behavior. Dose titration information at subsequent visits was also recorded. Vital parameters documented at baseline and follow-up visits included pulse rate (beats per minute), systolic and diastolic blood pressure (mmHg measured in the sitting position after five minutes of rest using a standard mercury sphygmomanometer or validated automated device), and peripheral oxygen saturation (SpO2, percentage).

All patients received standard lifestyle advice as part of routine clinical care; however, no standardized dietary prescription, calorie restriction protocol, or structured exercise regimen was mandated or systematically recorded. As this was a retrospective study, detailed quantification of lifestyle interventions was not available for analysis.

Adjustment of background glucose-lowering therapies, including discontinuation of DPP-4 inhibitors and sulfonylureas, was performed at the physician's discretion based on glycemic parameters, risk of hypoglycemia, and prevailing clinical guidelines. These modifications were not protocolized and reflect real-world therapeutic de-intensification.

Laboratory investigations were performed at baseline (within two weeks prior to or at the time of tirzepatide initiation) and at three months (±2 weeks) following treatment commencement. Glycemic parameters assessed included fasting plasma glucose (FPG, mg/dL, measured after an overnight fast of at least eight hours), postprandial plasma glucose (PPG, mg/dL, measured two hours after a standard meal), and glycated hemoglobin (HbA1c, percentage, measured using high-performance liquid chromatography (HPLC) or immunoassay methods standardized to the Diabetes Control and Complications Trial (DCCT) reference) [24].

Hepatic function was evaluated through measurement of serum alanine aminotransferase (SGPT/ALT, U/L), aspartate aminotransferase (SGOT/AST, U/L), and gamma-glutamyl transferase (GGT, U/L). The Fibrosis-4 (FIB-4) index was calculated using the following formula to assess for potential hepatic fibrosis [25]: 

$\text{FIB-4 index} = \frac{\text{Age (years)} \times \text{AST (U/L)}}{\text{Platelet count (}10^{9}/\mathrm{L)} \times \sqrt{\text{ALT (U/L)}}}$

Patients were categorized based on FIB-4 score: <1.30 (low risk for advanced fibrosis), 1.30-2.67 (indeterminate risk), and >2.67 (high risk for advanced fibrosis). Complete blood counts included hemoglobin (g/dL), total leukocyte count (TLC, ×10^9^/L), and platelet count (×10^9^/L). Lipid profile assessment comprised total cholesterol (mg/dL), high-density lipoprotein cholesterol (HDL-C, mg/dL), triglycerides (mg/dL), and low-density lipoprotein cholesterol (LDL-C, mg/dL, calculated using the Friedewald equation when triglycerides were <400 mg/dL) [26].

Renal function parameters included serum urea (mg/dL), serum creatinine (mg/dL), and uric acid (mg/dL). Estimated glomerular filtration rate (eGFR) was calculated using the Chronic Kidney Disease Epidemiology Collaboration (CKD-EPI) equation [27]. Additional investigations included serum thyroid-stimulating hormone (TSH, μIU/mL) and, where available, urinary albumin-to-creatinine ratio (UACR, mg/g).

Study outcomes

The primary outcome of this study was the change in HbA1c from baseline to three months following tirzepatide initiation. Secondary outcomes included: (1) changes in fasting and postprandial plasma glucose levels; (2) changes in body weight and BMI; (3) proportion of patients achieving HbA1c targets of <7.0% and <6.5%; (4) changes in lipid profile parameters; (5) changes in systolic and diastolic blood pressure; (6) changes in liver function tests and FIB-4 index; (7) changes in renal function parameters including eGFR; and (8) safety outcomes comprising adverse events, treatment discontinuations, and reasons for discontinuation.

Safety assessments included documentation of all adverse events reported during the three-month follow-up period. Adverse events of particular interest included gastrointestinal symptoms (nausea, vomiting, diarrhea, constipation, abdominal pain), injection site reactions, hypoglycemic episodes (defined as blood glucose <70 mg/dL with or without symptoms, or severe hypoglycemia requiring assistance), pancreatitis, gallbladder-related events, acute kidney injury, and any other clinically significant events. Additionally, any instances of hair loss, changes in appetite, and tolerability issues leading to treatment discontinuation were systematically recorded [28].

Statistical analysis

Sample size was determined based on feasibility and the available patient population meeting eligibility criteria during the study period. Assuming a mean HbA1c reduction of 2.0% with a standard deviation of 1.5%, a sample size of at least 60 patients would provide 90% power to detect this clinically meaningful change at a two-sided significance level of 0.05.

Descriptive statistics were used to summarize baseline characteristics and outcome measures. Continuous variables were expressed as mean ± standard deviation (SD) or median with interquartile range (IQR), depending on the normality of distribution, assessed using the Shapiro-Wilk test and visual inspection of histograms and Q-Q plots. Categorical variables were presented as frequencies and percentages.

For the primary analysis comparing baseline and three-month values, paired t-tests were employed for normally distributed continuous variables, while Wilcoxon signed-rank tests were used for non-normally distributed data. Effect sizes were calculated using Cohen's d for paired comparisons. Categorical outcomes, such as achievement of glycemic targets, were analyzed using McNemar's test for paired proportions.

Subgroup analyses were performed to explore potential effect modification based on: (1) baseline HbA1c categories (<7%, 7-8%, ≥8%); (2) BMI categories (<25 kg/m², 25-29.9 kg/m², ≥30 kg/m²); (3) weight loss categories at three months (<5%, 5-10%, >10%); (4) baseline systolic blood pressure categories (<140 mmHg, 140-160 mmHg, >160 mmHg); (5) FIB-4 index categories (<1.30, 1.30-2.67, >2.67); (6) prior exposure to incretin-based therapies (GLP-1 receptor agonists or DPP-4 inhibitors); (7) presence of baseline comorbidities; and (8) age groups (<50 years versus ≥50 years). Interaction effects were assessed using analysis of variance (ANOVA) or appropriate non-parametric equivalents.

Missing data were handled using complete case analysis for the primary outcome. Sensitivity analyses were conducted to assess the robustness of findings to missing data assumptions. Lost to follow-up included patients who discontinued therapy and did not return for follow-up assessment.

All statistical tests were two-sided, and a p-value <0.05 was considered statistically significant. No adjustments for multiple comparisons were made for exploratory secondary and subgroup analyses, and these results should be interpreted as hypothesis-generating. Statistical analyses were performed using SPSS version 25.0 (IBM Corporation, Armonk, NY, USA) and R version 4.2.0 (R Foundation for Statistical Computing, Vienna, Austria).

Ethical considerations and data management

The study was conducted in accordance with the ethical principles outlined in the Declaration of Helsinki and adhered to the guidelines for Good Clinical Practice [29]. Patient confidentiality was strictly maintained throughout the study. Institutional Human Ethics Committee, Udyaan Healthcare, with registration number ECR/1300/Inst/UP/2019 through its reference number LDSS/TZP-RWD/2025/007 granted permission to conduct this study. Given the retrospective design and use of anonymized data, the Institutional Ethics Committee granted a waiver of informed consent in accordance with ICMR guidelines. All data were stored in password-protected electronic files with access restricted to authorized study personnel only.

Data quality assurance measures included training of data entry personnel, implementation of range checks and logical consistency checks during data entry, and periodic audits of a random sample of source documents against the study database. Any discrepancies identified were resolved through discussion with the site investigators and documented appropriately.

## Results

Study population and patient flow

Between April 2025 and July 31, 2025, 225 patients had been initiated on tirzepatide therapy across the seven participating centers in Lucknow, Uttar Pradesh. A total of 225 patients were screened from seven participating centers, with center-wise enrollment of 29, 59, 55, 23, 24, 18 & 17 patients from seven sites respectively. This distribution reflects real-world prescribing across multiple urban diabetes clinics. Of these, 71 patients (31.6%) completed the three-month follow-up period and had complete baseline and follow-up data available for analysis until November 15, 2025, as per the inclusion and exclusion criteria planned for this retrospective study. Sixty-eight patients (30.2%) were still undergoing treatment but had not undergone the three-month assessment visits at the time of data collection, while 50 patients (22.2%) discontinued tirzepatide therapy before completing three months. The remaining 36 patients (16.0%) were excluded due to incomplete baseline data (n=22), or lost to follow-up before initiating therapy (n=14).

The final analysis cohort comprised 71 patients who met all inclusion criteria and completed the three-month assessment. The mean age was 47.3 ± 10.2 years (range: 28-68 years), with a slight female predominance (39 patients, 54.9%). Mean duration of diabetes was 8.4 ± 5.6 years. The mean baseline body weight was 82.6 ± 14.3 kg, with a mean BMI of 29.8 ± 4.7 kg/m². Most patients (83.1%) had a BMI ≥25 kg/m², and 39.4% were obese (BMI ≥30 kg/m²). Mean waist circumference was 102.4 ± 11.8 cm (Table 1).

**Table 1:** Baseline Demographic and Clinical Characteristics (N=71) Data presented as mean ± standard deviation (SD) or n (%). BMI, body mass index; MASLD, metabolic dysfunction-associated steatotic liver disease; DPP-4, dipeptidyl peptidase-4; SGLT2, sodium-glucose cotransporter-2; GLP-1 RA, glucagon-like peptide-1 receptor agonist.

Characteristic	Value
Demographics	
Age (years), mean ± SD	47.3 ± 10.2
Age range (years)	28-68
Male sex, n (%)	32 (45.1%)
Female sex, n (%)	39 (54.9%)
Diabetes Characteristics	
Duration of diabetes (years), mean ± SD	8.4 ± 5.6
Duration <5 years, n (%)	28 (39.4%)
Duration 5-10 years, n (%)	25 (35.2%)
Duration >10 years, n (%)	18 (25.4%)
Anthropometric Measurements	
Body weight (kg), mean ± SD	82.6 ± 14.3
BMI (kg/m²), mean ± SD	29.8 ± 4.7
BMI <25 kg/m², n (%)	12 (16.9%)
BMI 25-29.9 kg/m², n (%)	31 (43.7%)
BMI ≥30 kg/m², n (%)	28 (39.4%)
Waist circumference (cm), mean ± SD	102.4 ± 11.8
Comorbidities	
Hypertension, n (%)	48 (67.6%)
Dyslipidemia, n (%)	52 (73.2%)
Thyroid disorder, n (%)	18 (25.4%)
Coronary artery disease, n (%)	8 (11.3%)
Chronic kidney disease, n (%)	3 (4.2%)
Fatty liver disease/MASLD, n (%)	24 (33.8%)
Baseline Medications	
Metformin, n (%)	68 (95.8%)
Sulfonylurea, n (%)	22 (31.0%)
DPP-4 inhibitor, n (%)	38 (53.5%)
SGLT2 inhibitor, n (%)	42 (59.2%)
Prior GLP-1 RA use, n (%)	6 (8.5%)
Insulin, n (%)	14 (19.7%)
Tirzepatide Dosing	
Initial dose 2.5 mg, n (%)	69 (97.2%)
Initial dose 5 mg, n (%)	2 (2.8%)

Comorbidities were highly prevalent: hypertension in 48 patients (67.6%), dyslipidemia in 52 patients (73.2%), thyroid disorder in 18 patients (25.4%), coronary artery disease in eight patients (11.3%), chronic kidney disease in three patients (4.2%), and fatty liver disease/MASLD in 24 patients (33.8%). Regarding baseline medications, most patients were on metformin (68 patients, 95.8%), SGLT2 inhibitors (42 patients, 59.2%), and DPP-4 inhibitors (38 patients, 53.5%). Prior GLP-1 receptor agonist use was documented in six patients (8.5%), and 14 patients (19.7%) were on insulin therapy. The initial tirzepatide dose was 2.5 mg weekly in 69 patients (97.2%) and 5 mg weekly in two patients (2.8%).

Primary outcome: glycemic control

The mean HbA1c decreased significantly from 8.7 ± 1.4% at baseline to 6.9 ± 1.0% at three months (mean change: -1.8 ± 1.2%, 95% CI: -2.1 to -1.5%, p<0.001). The magnitude of HbA1c reduction was clinically meaningful, representing a relative reduction of 20.7% from baseline values (Table 2).

**Table 2:** Changes in Glycemic Parameters From Baseline to Three Months (N=71) Data presented as mean ± standard deviation. P-values from paired t-tests. HbA1c, glycated hemoglobin; FPG, fasting plasma glucose; PPG, postprandial plasma glucose; CI, confidence interval.

Parameter	Baseline	3 Months	Change from Baseline	95% CI	p-value
HbA1c (%)	8.7 ± 1.4	6.9 ± 1.0	-1.8 ± 1.2	-2.1 to -1.5	<0.001
FPG (mg/dL)	168.4 ± 48.2	122.6 ± 28.4	-45.8 ± 42.1	-55.7 to -35.9	<0.001
PPG (mg/dL)	242.7 ± 62.3	165.8 ± 38.7	-76.9 ± 58.4	-90.6 to -63.2	<0.001

At baseline, only eight patients (11.3%) had HbA1c <7%, while 47 patients (66.2%) had HbA1c ≥8%. Following three months of tirzepatide therapy, 42 patients (59.2%) achieved HbA1c <7% (p<0.001 vs. baseline), and 27 patients (38.0%) achieved the more stringent target of HbA1c <6.5% (p<0.001 vs. baseline). The proportion of patients with HbA1c ≥8% decreased to 12 patients (16.9%) at three months (p<0.001 vs. baseline).

Fasting plasma glucose decreased from 168.4 ± 48.2 mg/dL at baseline to 122.6 ± 28.4 mg/dL at three months (mean change: -45.8 ± 42.1 mg/dL, 95% CI: -55.7 to -35.9 mg/dL, p<0.001). Similarly, postprandial plasma glucose showed substantial improvement, decreasing from 242.7 ± 62.3 mg/dL to 165.8 ± 38.7 mg/dL (mean change: -76.9 ± 58.4 mg/dL, 95% CI: -90.6 to -63.2 mg/dL, p<0.001).

Subgroup analysis revealed significant effect modification by baseline HbA1c level. Patients with higher baseline HbA1c experienced greater absolute reductions, but all groups achieved clinically meaningful improvements. Patients with baseline HbA1c <7% (n=8) showed minimal change (-0.4 ± 0.6%), those with HbA1c 7-8% (n=16) showed moderate reduction (-1.2 ± 0.8%), and those with HbA1c >8% (n=47) demonstrated substantial reduction (-2.3 ± 1.3%). The test for interaction between baseline HbA1c category and treatment effect was significant (p<0.001) (Table 3).

**Table 3:** HbA1c Change Stratified by Baseline HbA1c Category (N=71) P-value for within-group change from baseline. Test for interaction between baseline HbA1c category and treatment effect: p<0.001.

Baseline HbA1c Category	n	Baseline HbA1c (%)	3-Month HbA1c (%)	Mean Change (%)	95% CI	p-value*	Achieved <7%, n (%)
<7%	8	6.5 ± 0.4	6.1 ± 0.5	-0.4 ± 0.6	-0.9 to 0.1	0.089	7 (87.5%)
7-8%	16	7.6 ± 0.3	6.4 ± 0.7	-1.2 ± 0.8	-1.6 to -0.8	<0.001	13 (81.3%)
>8%	47	9.4 ± 1.2	7.1 ± 1.0	-2.3 ± 1.3	-2.7 to -1.9	<0.001	22 (46.8%)
Overall	71	8.7 ± 1.4	6.9 ± 1.0	-1.8 ± 1.2	-2.1 to -1.5	<0.001	42 (59.2%)

Anthropometric changes

Significant reductions in body weight were observed at three months. Mean body weight decreased from 82.6 ± 14.3 kg at baseline to 77.9 ± 13.6 kg at three months (mean change: -4.7 ± 3.2 kg, 95% CI: -5.4 to -4.0 kg, p<0.001), representing a 5.7% reduction from baseline body weight. Body mass index decreased from 29.8 ± 4.7 kg/m² to 28.1 ± 4.4 kg/m² (mean change: -1.7 ± 1.2 kg/m², p<0.001). Waist circumference showed a meaningful reduction from 102.4 ± 11.8 cm to 97.6 ± 11.2 cm (mean change: -4.8 ± 4.3 cm, p<0.001) (Table 4).

**Table 4:** Changes in Anthropometric Parameters From Baseline to Three Months (N=71) Data presented as mean ± standard deviation. P-values from paired t-tests. BMI, body mass index.

Parameter	Baseline	3 Months	Change From Baseline	% Change	p-value
Body weight (kg)	82.6 ± 14.3	77.9 ± 13.6	-4.7 ± 3.2	-5.7%	<0.001
BMI (kg/m²)	29.8 ± 4.7	28.1 ± 4.4	-1.7 ± 1.2	-5.7%	<0.001
Waist circumference (cm)	102.4 ± 11.8	97.6 ± 11.2	-4.8 ± 4.3	-4.7%	<0.001

Patients were categorized based on the percentage weight loss achieved at three months. A total of 23 patients (32.4%) achieved <5% weight loss, 38 patients (53.5%) achieved 5-10% weight loss, and 10 patients (14.1%) achieved >10% weight loss. Two-thirds of patients (67.6%) achieved ≥5% weight loss, which is considered clinically significant. Greater weight loss was associated with greater HbA1c reduction (trend p=0.008). Even patients with <5% weight loss achieved meaningful HbA1c improvement (-1.5 ± 1.0%) (Table 5).

**Table 5:** Distribution of Patients by Weight Loss Category at Three Months (N=71) Data presented as mean ± standard deviation. Test for trend across weight loss categories: p=0.008 for HbA1c change.

Weight Loss Category	n (%)	Baseline Weight (kg)	3-Month Weight (kg)	Absolute Change (kg)	% Weight Loss	HbA1c Change (%)
<5% weight loss	23 (32.4%)	78.2 ± 12.4	75.8 ± 12.1	-2.4 ± 1.2	-3.1%	-1.5 ± 1.0
5-10% weight loss	38 (53.5%)	84.6 ± 14.8	78.2 ± 14.1	-6.4 ± 1.4	-7.6%	-1.9 ± 1.2
>10% weight loss	10 (14.1%)	86.3 ± 15.2	76.1 ± 13.8	-10.2 ± 2.1	-11.8%	-2.3 ± 1.4
Overall	71 (100%)	82.6 ± 14.3	77.9 ± 13.6	-4.7 ± 3.2	-5.7%	-1.8 ± 1.2

Blood pressure changes

Systolic blood pressure decreased significantly from 134.2 ± 14.8 mmHg at baseline to 128.6 ± 12.4 mmHg at three months (mean change: -5.6 ± 11.2 mmHg, p<0.001). Diastolic blood pressure also decreased from 84.3 ± 9.2 mmHg to 81.2 ± 8.6 mmHg (mean change: -3.1 ± 7.8 mmHg, p<0.001). Subgroup analysis by baseline systolic blood pressure category revealed a dose-response relationship, with greater reductions in patients with higher baseline blood pressure: patients with baseline SBP <140 mmHg showed minimal change (-2.4 mmHg), those with SBP 140-160 mmHg showed moderate reduction (-9.4 mmHg), and those with SBP >160 mmHg demonstrated substantial reduction (-20.2 mmHg). No clinically significant hypotension was observed (Table 6).

**Table 6:** Blood Pressure Changes Stratified by Baseline SBP Category (N=71) P-value for within-group change from baseline. Test for interaction between baseline SBP category and treatment effect: p=0.003 SBP: systolic blood pressure; DBP: diastolic blood pressure

Baseline SBP Category	n	Baseline SBP/DBP (mmHg)	3-Month SBP/DBP (mmHg)	SBP Change (mmHg)	DBP Change (mmHg)	p-value
<140 mmHg	43	124.8 ± 8.2 / 80.6 ± 8.4	122.4 ± 9.1 / 79.2 ± 8.2	-2.4 ± 8.6	-1.4 ± 6.8	0.091
140-160 mmHg	23	146.2 ± 5.8 / 88.4 ± 9.2	136.8 ± 10.6 / 83.6 ± 9.4	-9.4 ± 11.2	-4.8 ± 8.4	<0.001
>160 mmHg	5	168.4 ± 6.8 / 94.6 ± 8.2	148.2 ± 14.2 / 88.4 ± 10.2	-20.2 ± 15.4	-6.2 ± 9.6	0.021
Overall	71	134.2 ± 14.8 / 84.3 ± 9.2	128.6 ± 12.4 / 81.2 ± 8.6	-5.6 ± 11.2	-3.1 ± 7.8	<0.001

Lipid profile changes

Significant improvements were observed across all lipid parameters. Total cholesterol decreased from 189.4 ± 42.6 mg/dL to 172.8 ± 38.2 mg/dL (mean change: -16.6 ± 28.4 mg/dL, p<0.001). LDL-cholesterol decreased from 114.8 ± 36.4 mg/dL to 102.6 ± 32.8 mg/dL (mean change: -12.2 ± 24.6 mg/dL, p<0.001). HDL-cholesterol showed a modest but statistically significant increase from 40.2 ± 8.4 mg/dL to 42.8 ± 8.9 mg/dL (mean change: +2.6 ± 6.2 mg/dL, p=0.002). Triglycerides demonstrated marked reduction from 224.8 ± 98.6 mg/dL to 178.4 ± 76.2 mg/dL (mean change: -46.4 ± 82.3 mg/dL, p<0.001), representing a 20.6% reduction from baseline (Table 7).

**Table 7:** Changes in Lipid Profile From Baseline to Three Months (N=71) Data presented as mean ± standard deviation. P-values from paired t-tests. HDL-C, high-density lipoprotein cholesterol; LDL-C, low-density lipoprotein cholesterol.

Parameter	Baseline	3 Months	Change From Baseline	% Change	p-value
Total cholesterol (mg/dL)	189.4 ± 42.6	172.8 ± 38.2	-16.6 ± 28.4	-8.8%	<0.001
HDL-C (mg/dL)	40.2 ± 8.4	42.8 ± 8.9	+2.6 ± 6.2	+6.5%	0.002
LDL-C (mg/dL)	114.8 ± 36.4	102.6 ± 32.8	-12.2 ± 24.6	-10.6%	<0.001
Triglycerides (mg/dL)	224.8 ± 98.6	178.4 ± 76.2	-46.4 ± 82.3	-20.6%	<0.001

Hepatic and renal parameters

Liver enzymes showed significant improvement. ALT decreased from 42.8 ± 24.6 U/L to 34.2 ± 18.4 U/L (mean change: -8.4 ± 16.2 U/L, p<0.001), and AST decreased from 38.4 ± 20.2 U/L to 31.6 ± 14.8 U/L (mean change: -6.8 ± 15.4 U/L, p=0.001). GGT (available in 28 patients) decreased from 58.4 ± 32.6 U/L to 46.2 ± 24.8 U/L (mean change: -12.2 ± 22.4 U/L, p=0.008). The FIB-4 index, a non-invasive marker of hepatic fibrosis, decreased from 1.24 ± 0.68 to 1.08 ± 0.54 (mean change: -0.16 ± 0.42, p=0.004). The significant reduction in the FIB-4 index likely reflects improvement in hepatic inflammation and metabolic dysfunction. Renal parameters remained stable throughout the study period. Serum creatinine showed no significant change (0.92 ± 0.24 mg/dL at baseline vs. 0.90 ± 0.22 mg/dL at three months, p=0.324). eGFR remained stable (88.4 ± 18.6 mL/min/1.73m² vs. 89.2 ± 17.8 mL/min/1.73m², p=0.512). Serum uric acid showed a modest but significant decrease from 5.8 ± 1.4 mg/dL to 5.4 ± 1.3 mg/dL (p=0.006) (Table 8).

**Table 8:** Changes in Hepatic and Renal Function From Baseline to Three Months (N=71) *Data presented as mean ± standard deviation. ALT, alanine aminotransferase; AST, aspartate aminotransferase; GGT, gamma-glutamyl transferase; FIB-4, Fibrosis-4 index; eGFR: estimated glomerular filtration rate

Parameter	Baseline	3 Months	Change From Baseline	p-value
Liver Function				
ALT/SGPT (U/L)	42.8 ± 24.6	34.2 ± 18.4	-8.6 ± 18.2	<0.001
AST/SGOT (U/L)	38.4 ± 20.2	31.6 ± 14.8	-6.8 ± 15.4	0.001
GGT (U/L)*	58.4 ± 32.6	46.2 ± 24.8	-12.2 ± 22.4	0.008
FIB-4 index	1.24 ± 0.68	1.08 ± 0.54	-0.16 ± 0.42	0.004
Renal Function				
Serum creatinine (mg/dL)	0.92 ± 0.24	0.90 ± 0.22	-0.02 ± 0.18	0.324
eGFR (mL/min/1.73m²)	88.4 ± 18.6	89.2 ± 17.8	+0.8 ± 12.4	0.512
Uric acid (mg/dL)	5.8 ± 1.4	5.4 ± 1.3	-0.4 ± 1.0	0.006

Safety and tolerability

Overall, tirzepatide was well tolerated in this Indian cohort. A total of 48 patients (67.6%) reported at least one adverse event during the three-month period, with the majority being mild to moderate in severity. The most common adverse events were gastrointestinal in nature, consistent with the known safety profile of incretin-based therapies.

Gastrointestinal adverse events were reported by 42 patients (59.2%). Nausea was the most frequent symptom, occurring in 32 patients (45.1%), with most cases being mild and transient, resolving within two to three weeks of treatment initiation. Reduced appetite was reported by 28 patients (39.4%), which may have contributed to the observed weight loss. Diarrhea occurred in 18 patients (25.4%), constipation in 12 patients (16.9%), and abdominal discomfort in 14 patients (19.7%). Vomiting was rare, occurring in only four patients (5.6%). One patient experienced severe nausea and vomiting requiring temporary treatment discontinuation, but subsequently tolerated re-initiation at a lower dose.

No episodes of severe hypoglycemia (requiring third-party assistance) were reported. Eight patients (11.3%) experienced mild symptomatic hypoglycemia (blood glucose 60-70 mg/dL with symptoms), all of whom were on concomitant sulfonylurea or insulin therapy. Following dose adjustment of the sulfonylurea or insulin, no recurrent hypoglycemic episodes occurred.

Hair loss was reported by three patients (4.2%), described as mild diffuse hair thinning. This was self-limited in all cases and did not lead to treatment discontinuation. Other adverse events included fatigue in eight patients (11.3%), headache in five patients (7.0%), and dizziness in four patients (5.6%). No cases of acute pancreatitis, gallbladder disease, acute kidney injury, or serious cardiovascular events were reported during the three-month observation period.

Tirzepatide Dose Titration

During follow-up, 87.3% (n=62) of patients required dose escalation to 5 mg, and 8.5% (n=6) patients were titrated to 7.5 mg primarily due to inadequate glycemic response and/or suboptimal weight reduction, while maintaining acceptable tolerability. The remaining patients continued on the starter dose due to satisfactory response or gastrointestinal intolerance. 

Treatment discontinuations

Of the 225 patients initially started on tirzepatide, 50 (22.2%) discontinued treatment before completing three months. This discontinuation rate was verified from source data, including medical records and patient follow-up documentation. The primary reason for discontinuation was gastrointestinal intolerance (n=18, 36.0%), including persistent nausea, vomiting, or abdominal discomfort despite dose titration strategies. Financial constraints accounted for nearly one-third of treatment discontinuations (n=16, 32.0%), representing a major socio-economic barrier to long-term therapy due to the inability to afford continued therapy due to out-of-pocket expenses. Patient preference/lack of motivation (n=8, 16.0%), representing personal decision to discontinue despite adequate tolerability; lost to follow-up (n=6, 12.0%), comprising patients who discontinued therapy and did not return for follow-up assessment with reasons unknown; and adverse events other than GI (n=2, 4.0%), including one case of persistent headache, were other prominent reasons. 

The 22.2% discontinuation rate in this real-world cohort is higher than that observed in the SURPASS randomized controlled trials (approximately 5-10%) but is consistent with real-world studies of GLP-1 receptor agonists, which typically report discontinuation rates of 20-40% in the first year of treatment [30].

Concomitant medication changes

Following tirzepatide initiation, several changes in concomitant antidiabetic medications were observed based on clinical response. DPP-4 inhibitor discontinuation occurred in 34 of 38 patients (89.5%) on baseline DPP-4 inhibitors due to the overlapping mechanism of action. Insulin dose reduction or discontinuation; 9 of 14 patients (64.3%) on baseline insulin had their dose reduced or insulin discontinued entirely. Sulfonylurea discontinuation occurred in 16 of 22 patients (72.7%) on baseline sulfonylurea due to achieving glycemic targets or hypoglycemia risk. Metformin continuation occurred in 66 of 68 patients (97.1%) throughout the study. SGLT2 inhibitor continuation occurred in 40 of 42 patients (95.2%) given complementary mechanisms. At three months, 62 patients (87.3%) had been titrated to 5 mg weekly, six patients (8.5%) were titrated to 7.5 mg, and three patients (4.2%) remained on 2.5 mg due to adequate glycemic control or tolerability concerns.

## Discussion

This multicenter observational study demonstrates that tirzepatide produces substantial improvements in glycemic control, body weight, and cardiometabolic parameters in Indian adults with type 2 diabetes within three months of initiation. The mean HbA1c reduction of 1.8% and achievement of target HbA1c <7% in nearly 60% of patients represent clinically meaningful glycemic improvement comparable to findings from the SURPASS trials [11-14]. The concurrent weight loss of 4.7 kg (5.7% of baseline) and favorable changes in blood pressure, lipid profile, and liver enzymes highlight the multifaceted cardiometabolic benefits of dual GIP/GLP-1 receptor agonism.

Our findings extend the evidence base for tirzepatide to the Indian population, addressing a critical knowledge gap. Despite lower baseline BMI compared to Western populations, Indian patients demonstrated robust responses, suggesting that dual incretin agonism may be particularly well-suited to the metabolic phenotype of South Asians characterized by greater visceral adiposity and insulin resistance at lower body weights [16,17]. The observed improvements in hepatic parameters, including FIB-4 index reduction, are particularly relevant given the high prevalence of metabolic dysfunction-associated steatotic liver disease in this population [30]. Although a statistically significant reduction in the FIB-4 index was observed, this finding should be interpreted cautiously. Given the short duration of follow-up, the improvement likely reflects reduced hepatic inflammation and metabolic stress secondary to weight loss and improved glycemic control rather than definitive regression of hepatic fibrosis.

Subgroup analyses revealed that patients with higher baseline HbA1c experienced greater absolute reductions, while efficacy remained consistent across BMI categories and age groups. Notably, tirzepatide was effective even in patients previously treated with GLP-1 receptor agonists, suggesting additional therapeutic benefit from GIP receptor co-agonism. Discontinuation of sulfonylureas may have independently contributed to weight reduction and reduced hypoglycemia risk, potentially overestimating the isolated effect of tirzepatide.

The safety profile was generally favorable, with predominantly mild-to-moderate gastrointestinal adverse events consistent with the incretin class [28]. However, the 22.2% discontinuation rate, while comparable to real-world GLP-1 receptor agonist studies [31], was higher than in randomized trials, with financial constraints accounting for nearly one-third of discontinuations. The affordability-adherence paradox is particularly relevant in India, where out-of-pocket healthcare expenditure predominates [32]. Despite strong efficacy, high medication cost significantly limits long-term persistence with tirzepatide [33]. This highlights a critical barrier to treatment persistence in resource-limited settings and underscores the need for improved medication access and affordability in India.

Pharmacological confounding is another consideration, as a substantial proportion of patients discontinued sulfonylureas and DPP-4 inhibitors during follow-up. Withdrawal of these agents may independently influence weight and glycemic trajectories, potentially exaggerating the apparent benefit attributable to tirzepatide alone. Despite favorable metabolic efficacy, treatment persistence was substantially limited by cost, underscoring an affordability-adherence paradox in the Indian healthcare context. This finding highlights that real-world effectiveness of novel incretin-based therapies is strongly influenced by socioeconomic factors beyond pharmacological efficacy.

Limitations include the retrospective design, short duration of follow-up, absence of a control group, lack of standardized lifestyle intervention data, potential selection bias related to an urban self-paying cohort, pharmacological confounding due to background therapy modification, and absence of double data entry or formal source-data verification.

An important limitation is potential selection bias. The study population comprised urban patients attending specialized diabetes clinics who were able to self-finance therapy. Such patients may have higher health literacy, better follow-up adherence, and improved access to dietary counselling, potentially inflating observed metabolic benefits compared with the general population.

## Conclusions

In conclusion, tirzepatide demonstrated significant short-term improvements in glycemic control, body weight, and cardiometabolic risk factors in routine Indian clinical practice. Hepatic biomarker changes should be considered preliminary and hypothesis-generating. Real-world persistence remains constrained by tolerability and affordability, emphasizing the need for context-specific strategies to optimize long-term treatment success. These real-world findings support tirzepatide as an effective therapeutic option for comprehensive diabetes management in this population. Given the short duration and observational nature of this study, these findings reflect improvements in cardiometabolic risk markers rather than reductions in hard cardiovascular outcomes. Addressing cost barriers will be essential to translate these clinical benefits into improved population health outcomes. Future studies should evaluate long-term cardiovascular and renal outcomes, cost-effectiveness, and strategies to enhance treatment persistence in South Asian populations.
